# Effective Reduction of Transgene‐Specific Immune Response With rAAV Vectors Co‐Expressing miRNA‐UL112‐5p or ERAP1 shRNA

**DOI:** 10.1111/jcmm.70308

**Published:** 2025-01-17

**Authors:** Xiaoping Huang, Xiao Wang, Yaqi Sun, Xinrui Xie, Luming Xiao, Yihang Xu, Qiongshi Yan, Xianxiang Xu, Ling Li, Wentao Xu, Wenting Weng, Wenlin Wu, Xiaolan Xie, Congjie Dai, Yong Diao

**Affiliations:** ^1^ College of Chemical Engineering and Materials Sciences Quanzhou Normal University Quanzhou China; ^2^ Institute of Molecular Medicine Huaqiao University Quanzhou China; ^3^ College of Marine and Food Science Quanzhou Normal University Quanzhou China; ^4^ Fujian Province Key Laboratory for the Development of Bioactive Material From Marine Algae Quanzhou China

**Keywords:** ERAP1, immunity, miRNA‐UL‐112‐5p, rAAV

## Abstract

Recombinant adeno‐associated virus (rAAV) has emerged as one of the best gene delivery vectors for human gene therapy in vivo. However, the clinical efficacy of rAAV gene therapy is often hindered by the host immune response against its transgene products. Endoplasmic reticulum aminopeptidase 1 (ERAP1) is specialised to process peptides presented by class I molecules of major histocompatibility complex. Therefore, we hypothesise that modulation of the ERAP1 activity in rAAV transduced cells may be favoured to evade immune response against transgene products. In this study, we incorporated either miRNA‐UL112‐5p or ERAP1 shRNA into rAAV vectors expressing full‐length ovalbumin (OVA) as a model antigen, and evaluated their effects for antigen presentation, cellular and humour immune response induced by OVA expression. The results indicated that silencing ERAP1 using miR‐UL112‐5p or ERAP1 shRNA did not affect the expression of OVA in cells, but inhibited the processing and presentation of OVA antigen peptide SIINFEKL in antigen presenting cells (APCs). Moreover, the rAAV vector co‐expressing ERAP1 shRNA maintains stable and high expression of OVA in vivo, while simultaneously suppressing the humoral immunity of OVA. In addition, experimental results demonstrated that rAAV vectors incorporated ERAP1 shRNA efficiently repress costimulatory signals in dendritic cells (DCs), significantly attenuated the cytotoxic T‐cell response, allowed for sustained transgene expression and reduced clearance of transduced muscle cells in mice. Moreover, our study suggested that the incorporation of miRNA‐UL112‐5p or ERAP1 shRNA into rAAV vectors effectively reduced transgene products induced immune response. The proposed method may potentially be applied in clinics to deliver therapeutic proteins safely and efficiently.

## 1 Introduction

Recombinant adeno‐associated viral vectors (rAAV) are small, universal and non‐pathogenicity recombinant particles used in gene delivery to therapy genetic diseases. Wild‐type AAV particles have a diameter of 20–25 nm and contain a single‐stranded genome of approximately 4700 nucleotides. The genome of this virus is composed of two open reading frames, designated rep and cap, flanked by inverted terminal repeats. These elements contain all the necessary cis requirements for replication and packaging [1, 2]. The process of rAAV transduction is extremely intricate. It commences with receptor‐dependent endocytosis, following internalisation. Then, rAAV is transported through endosomal pathways. Finally, rAAV enters the nucleus through the nuclear pore complex. Once in the nucleus, the virus undergoes capsid uncoating to liberate the genome and express the transgene [3, 4]. The drug initially approved for gene treatment utilises rAAV1 gene delivery in the muscles in Europe [5]. Although rAAV is advantageous as a gene delivery system, many preclinical gene transfer trials using rAAV are restricted by transgene‐specific cellular immunity or humoral immunity, causing gene‐modified cell loss or tissue injury, based on recent reports [6, 7]. Some parameters, including transgene‐encoded protein nature, dosage, route and host immune resistance, may be associated with anticapsid immune responses [8]. During the last decade, rAAV has been widely applied to treat uncommon disorders in tissues including the brain, liver, heart, muscle and eye [9]. There are a total of six treatments receiving marketing approval from the European Medicines Agency (Glybera, Luxturna, Zolgenmsa, Upstaza, Roctavian) and the US Food and Drug Administration (Luxturna, Zolgensma) [10].

However, the loss of efficacy can be frequently observed in clinical studies, probably related to different factors, such as anti‐rAAV immunity [10, 11, 12]. Anti‐rAAV immunity includes pre‐existing antibodies, T‐cell immunity and complement system activation [13, 14, 15]. Cross‐presentation or classical presentation of the MHC‐I antigen can elicit a cytotoxic CD8+ T lymphocyte (CTL) response to the AAV2 capsid [12, 16]. Recently, one side effect of haemophilia gene treatment has been recently reported in a clinical study, which can be associated with the capsid‐specific CTL response, causing AAV‐mediated hepatocyte loss [11]. However, as suggested in many laboratories, capsid‐specific CTLs cannot eliminate AAV‐ transduced target cells from mice, regardless of the transgene type, vector administration route and promoter [17, 18, 19, 20]. Another recently published report indicated that CTL responses were triggered by cryptic epitopes produced in therapeutic genes. p18(APVLRDIDL), p31(SPVNQQCHF) and p52 (SPVLRLFFL) in the alternative reading frames of the F9 transgene triggered the reactivity of CD8+ T cells in B0702 transgenic mice expressing human major histocompatibility complex (MHC) class I molecule [17]. Preclinical research has suggested that rAAV vectors can consistently express numerous self and non‐self‐transgenes in various tissues, including muscle, liver, lung, heart, brain and eye [7]. Certainly, this is a challenge for the development of rAAV gene therapy. MHC class I molecules in the endoplasmic reticulum (ER) can bind to cellular and viral protein‐derived peptides, which are transported and displayed on the cell surface. In addition, this process assists CD8+ T cells to identify and eliminate virus‐infected cells or those with mutations [21, 22, 23]. Degradation by proteasomes into oligopeptides is the initial step in the formation of MHC‐binding peptides in many proteins [22, 24]. MHC class I can bind to peptides of 8–10 amino acids, which is determined by the unique structure of that MHC molecule, or more specifically, its allele [25]. ERAP1, a vital aminopeptidase, is responsible for trimming the ER‐targeting model NH2‐extended precursor to achieve antigen presentation. ERAP1 processes peptide precursors produced by proteasomes in the cytosol and trims peptides to suitable length for antigen presentation by MHC class I molecules [25].

Through specific ERAP1 silencing, decreasing the risk of CTL activation by virus‐derived pMHC I have been a means of immune evasion against various viruses. The evolution of HIV has led to mutations in the flanking regions of the dominant antigenic determinants of the CTL response, which can reduce the efficiency of ERAP1‐mediated antigen processing, thereby lowering the HIV‐induced CTL response [26]. In cervical cancer patients with persistent HPV infection and malignant transformation of cervical epithelial cells, cancer cell metastasis and decreased survival rates are associated with ERAP1 mutations [27]. Downregulation of ERAP1 activity causes reduced presentation of pathogenic antigens, and inability to effectively induce cellular immune rejection in patients, allowing tumour cells to continue to grow [28]. miR‐UL112‐5p in the human cytomegalovirus (HCMV) genome interferes with MHC I molecule‐mediated antigen presentation by targeting ERAP1 in host cells, affecting the immune performance of HCMV‐infected cells. The production of potential antigenic peptides causes CTL to fail to recognise viral antigens, causing immune escape [29].

In this study, we demonstrated that silencing ERAP1 reduced OVA antigen presentation at APC, therefore escaping cell‐mediated immunity by inhibiting cytotoxic CTLs. The OVA (257–264) peptide (SIINFEKL) is an H‐2Kb –restricted CTL epitope derived from chicken ovalbumin. Humoral immunity is shown to be less of a contributing factor once the CTL response is overcome and transgene secreted product levels are increased in circulation. The incorporation of ERAP1 shRNA can be an effective strategy to suppress immune responses when redosing with a different vector serotype expressing the same transgene products as the initial dose. The findings indicate that silencing ERAP1 using miRNA‐UL112‐5p or shRNA is a reliable method to overcome the immune response induced by transgene products. Therefore, it enhances the efficacy and safety of therapeutic gene delivery.

## 2 Methods

### 2.1 Vector Plasmid Establishment and rAAV Generation

Full‐length OVA or EGFP cDNA was cloned into a cis‐plasmid in the rAAV expression cassette from the CB promoter to the bovine growth hormone (BGH) polyA signal, aiming to generate pAAV‐CB‐OVA. ERAP1 shRNA (5′‐TCTTCTTGCTCAAATTACCTTGGCAAGTGTAAGGTAAT TTGAGCAAGAAGA‐3′) and pre‐miRNA‐UL112‐5p sequences were inserted into the U6 promoter. Sanger sequencing was performed to demonstrate the presence of the expression cassette.

Once the plasmid has been constructed, the production of rAAV follows the reported method [3, 30, 31]. Three plasmids were transfected into 293 T cells using linear polyethyleneimine with a molecular weight of 25,000 (PEI 25 K, Polysciences). Then, 72 h after transfection, cells were harvested, subjected to three freeze–thaw cycles and treated with benzylase (50 U/mL crude lysis buffer) for 60 min at 37°C. Subsequently, the viral vector is purified by caesium chloride gradient centrifugation. Viral vectors were concentrated by ultracentrifugation, washed three times with PBS and finally resuspended in PBS. Next, qPCR was used to detect the titre of the rAAV vector.

### 2.2 qPCR And RT‐qPCR

TRIzol reagent (Invitrogen, Carlsbad, CA) was used to isolate total tissue or cellular RNA, following the specific protocols. Mouse tissue DNA was isolated using the QIAamp Genomic DNA Kit (QIAGEN) based on the instructions of the manufacturer. The used forward quantitative PCR (qPCR) primers were as follows: Tumour necrosis factor α (TNF‐α) F: 5′‐GCTGGAG TGGCTGAGCCA‐3′′. R: 5′′‐TGAG GAGCACGTAGTCGG‐3′′; OVA F: 5′‐AATACTTGCAGTGTG TGAAG‐3′′. R: 5′‐TGGCTGAAGG A CATTTCTGA‐3′′

### 2.3 Mouse Experiments

The 6‐8‐week‐old male C57BL/6 mice were provided by the Shanghai Laboratory Animal Center (Chinese Academy of Sciences) and raised in an animal facility in a specific pathogen‐free environment. For the in vivo experiment, we administered intravenous injection or intramuscular injection of PBS or 2 × 10^11^ Vector Genomes(vg)/mouse of rAAV2‐OVA or rAAV2‐OVA‐ERAP1 shRNA into each mouse (*n* = 3/group). Afterwards, blood was sampled from the facial vein to obtain serum in BD Microtainer serum separator tubes.

### 2.4 Assessment of OVA Protein Expression and Anti‐OVA Antibody Level

ELISA was performed to analyse OVA and anti‐OVA IgG levels in the serum. The serum or culture supernatants were collected. Corresponding ELISA kits (Shanghai Ruifan Biotechnology, Shanghai, China) were employed to measure OVA and OVA IgG levels following the specific protocols. Briefly, 96‐well Corning Immunoplates (Corning, NY, USA) were coated with OVA protein or anti‐OVA antibody in 100 μL of PBS in each well. Subsequently, the plates were sealed and incubated overnight at room temperature. Next, all wells were aspirated and rinsed three times with 0.05% Tween‐20 in PBS (Washing Buffer). Subsequently, Reagent Diluent (1% BSA in PBS) was incubated for 2 h at room temperature. To determine OVA, ELISA diluent (Reagent Diluent) was added to dilute the samples to 100 folds, and OVA protein standards (Sigma‐Aldrich) were diluted 2 folds from 10 ng/mL. Afterwards, we introduced the sample or standard (100 μL) into the plates for 60 min at room temperature. The sample was washed four times, followed by addition of peroxidase‐labelled anti‐OVA antibody for 60 min of incubation at room temperature. To identify anti‐OVA IgG1 levels, the sample was diluted 1:200 with anti‐OVA IgG1 as the standard. At 60 min post‐incubation in OVA‐coated plates, each well was rinsed and incubated with HRP‐labelled anti‐mouse IgG1, followed by incubation for 60 min at room temperature. After washing four times, plates were incubated with 100 μL TMB HRP Substrate. A Tecan Microplate Reader (Tecan Infinite M200, Tecan Trading AG, Mannedorf, Switzerland) was used to measure absorbance at 450 nm.

### 2.5 In Vitro Transfection Experiments

Mouse myoblast C2C12 cells were transfected with OVA expression plasmids with or without miRNA‐UL112‐5p/ ERAP1 shRNA (Pricella, CL‐0044) using PolyJet transfection reagents (SignaGen Laboratories), according to the instructions of the manufacturer. Afterwards, C2C12 cells were cultured in Dulbecco's modified Eagle medium (Pricella, PM150210) containing 10% fetal bovine serum (FBS, Pricella, 164210) and 1% penicillin/streptomycin (Pricella, PB180120), while mouse dendritic JAWS II cells (ATCC, CRL‐11904) and human dendritic cells (Pricella, CP‐H179A) were cultured in α‐minimum essential medium (Pricella, PM150421) containing 10% FBS, 5 ng/mL murine GM‐CSF, 4 mM l‐glutamine and 1 mM sodium pyruvate. Nucleofection was performed to transfect JAWS II and human DCs. Subsequently, 2.0 × 10^6^ cells were harvested for resuspension in Nucleofector Solution (100 μL, Celetrix biotechnologies, SP100) at room temperature. After plasmid addition, the mixed sample was added to Nucleo‐cuvette Vessels, and the program was selected for immature mouse DCs (Celetrix). Nest, the medium (2 mL) was introduced to split the cells in a 24‐well plate (500 μL/well). At 3 days post‐transfection, the supernatant was used to perform OVA ELISA.

### 2.6 In Vivo CTL Assay

PBS, recombinant AAV2‐OVA, or recombinant AAV2‐OVA‐ERAP1 shRNA was delivered into 6‐8‐week‐old male C57BL/6 mice through the tail vein. At 17 days post‐injection, a 70‐μm Cell Strainer (BD Biosciences, 352,350) was used to mash spleens to separate splenocytes from naive C57BL/6 mice. Afterwards, red blood cells were lysed, and the suspension was centrifuged at 200 g for 3 min. RPMI‐1640 medium (Thermo Fisher Scientific, catalogue 22,400,089) was used for rinsing rest splenocyte cell pellets, and the splenocytes were suspended into sterile PBS, followed by labelling using high‐dose CFSE (CFSEhi, 5 μM), pulsing using the SIINFEKL peptide (OT‐1, 1 μg/mL) or labelling using low‐dose CFSE (CFSElo, 0.5 μM) without pulsing. Next, the target cell populations (1:1) were blended for intravenous cotransferred (4 × 10^7^ cells) into PBS‐ or rAAV2‐treated mice. After 6 h, flow cytometry was used to separate and analyse splenocytes to determine the proportion of the remaining target cells. The following formula was used to determine the specific killing efficiency: specific killing efficiency = (1‐ratio of control, unprimed recipient/ratio of primed recipient) × 100; the ratio was unpulsed CFSElo cell proportion/pulsed CFSEhi cell proportion.

### 2.7 Flow Cytometry

After suspension of cells in 100 μL PBS containing 1% FBS, the cells were blocked and probed with relevant antibodies at 4°C for 30 min. FITC–anti‐CD11c (catalogue bs‐20689R) and Phycoerythrin–anti‐CD86 (catalogue bs‐1035R) mAb were purchased from Bioss Biosciences. For tetramer staining, mouse blood in BD Microtainer tubes containing dipotassium EDTA was obtained. Then, iTAg MHC Tetramer H‐2Kb SIINFEKL‐PE (T03000, MBL) and FITC rat anti‐mouse CD8a mAb (Bioss, bs‐10699R) were introduced for 30 min of incubation at 4°C. Subsequently, sample processing was performed following the specific protocols. FACSCanto II (BD Biosciences) was used for the flow cytometry analysis. The FlowJo software was used to analyse data.

### 2.8 Tumour Growth Model

The 6‐8‐week‐old C57BL/6 mice were inoculated subcutaneously with 2 × 10^6^ E.G7‐OVA cells (Oricell, M5‐1001) via flanks. The following formula was used to determine tumour volume: tumour volume = 0.4 × length (mm) × width (mm). After reaching a tumour volume of 400 mm^3^, mice were euthanised by CO_2_ to collect and weigh the tumour tissues.

### 2.9 Immunohistochemistry (IHC)

Mouse tissues were immersed overnight in 10% formalin buffer (Biosharp, BL539A) before paraffin embedding. Then, tissues were prepared into 6‐μm sections before H&E staining. Images were obtained under an Olympus BX43 microscope. IHC was conducted in accordance with a previous description [32]. The primary antibody against CD8 (1:250, AG1414, Beyotime Technology) was added to stain the paraffin‐embedded muscle sections for 60 min. After washing with PBS, the primary antibody was detected using Alexa Fluor 488 secondary antibody (A0423, Beyotime Technology), following the specific protocols. In addition, a Zeiss LSM710 laser scanning confocal microscope was used for the image acquisition.

### 2.10 HeLa‐K^b^ and C2C12‐K^b^ Cell Lines

Lentivirus was used to obtain stable expression of H‐2K^b^ in HeLa and C1C12 cells. Cells were transfected with pLenti‐H‐2K^b^, pMD2G and psPAX2 plasmids (ratio: 3:2:1; 20 μg per 15‐cm plate). At 3 days post‐transfection, 0.45‐μm filters were used to filter the culture medium to remove debris and cells. Meanwhile, supernatants were centrifuged at 20,000 ×  *g* for 2 h to pellet the virus. Then, viruses were introduced into the HeLa and C1C12 cell media. At 3 days post‐infection, the cells were selected and exposed to 1 μg/mL puromycin. In addition, a limiting dilution was used to generate single‐cell subclones, followed by testing for stable expression using immunoblotting assays.

### 2.11 Statistical Analysis

All data are presented as mean ± SD. Unpaired student's *t*‐test (2‐tailed) and 2‐way ANOVA, with or without post hoc testing, were calculated using GraphPad Prism 8. Differences represented significance when *p* values were less than 0.05.

### 2.12 Study Approval

Experiments involving animal subjects followed the ARRIVE guidelines and the Guide for Care and Use of Animals in Research of the People's Republic of China. All the experimental protocols (no. AHQU019326) were authorised by the Huaqiao University (Quanzhou, China) Scientific Ethics Committee.

## 3 Results

### 3.1 ERAP1 Downregulation Does Not Affect rAAV Transgene Expression

**FIGURE 1:**
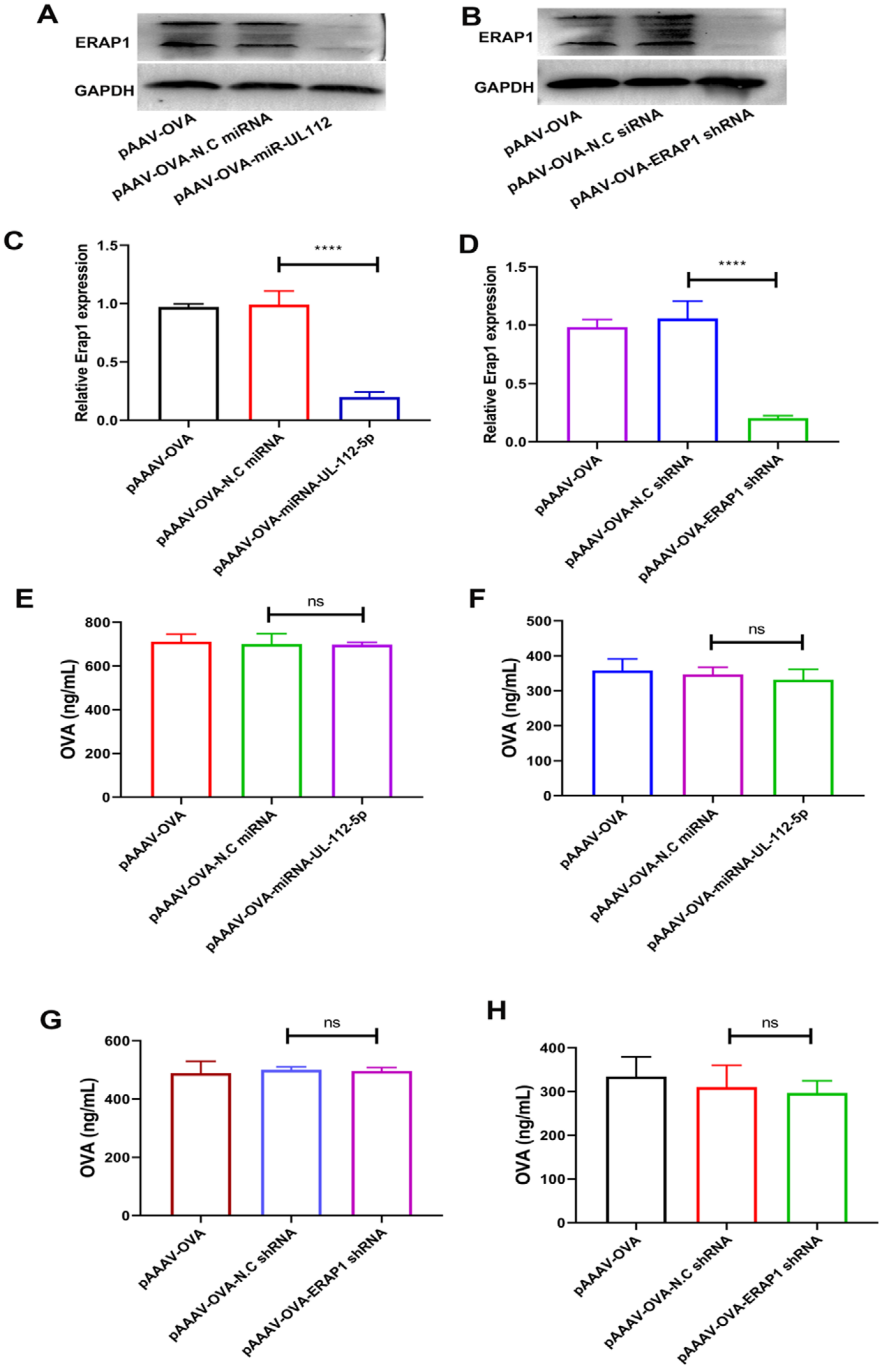
Effects of ERAP1 silencing on rAAV transgenes expression. (A, C) The expression of ERAP1 in HeLa cells after either Negative control miRNA (N.C miRNA) or miRNA‐UL112‐5p transfection was determined by Western‐blot assay. (B, D) ERAP1 levels in C1C12 cells transfected with either Negative control shRNA or ERAP1 shRNA were measured through Western‐blot. pAAV‐OVA, pAAV‐OVA‐miRNA‐UL112‐5p or pAAV‐OVA‐ERAP1 shRNA plasmid was transfected into HeLa (E), Human DC (F), C2C12 (G) or JAWSII (H) cells. At 72 h post‐transfection, supernatants were harvested to quantify OVA level through ELISA. The results are indicated by mean ± SD (*n* = 3). ****p < 0.0001; ^ns^ *p* > 0.05.

ERAP1 is a vital constituent of antigen processing and is responsible for trimming peptide precursors of suitable length for binding to MHC class I molecules; thus, antigenic epitopes can be recognised through CD8+ T cells. miR‐UL‐112‐5p, containing the 7‐nucleotide seed sequence matched with the human ERAP1 mRNA 3′‐untranslated region (3′UTR) at the 300–315 position (Figure S1A), was used to decrease human ERAP1 expression. To demonstrate whether miR‐UL112‐5p inhibited ERAP1 in HeLa cells, an ERAP1 3′‐UTR Luciferase Reporter Assay was performed. miR‐UL112‐5p transfection suppressed luciferase reporter gene expression, which was regulated by the ERAP1 3′UTR in the reporter plasmid, while scrambled miRNA exhibited no impact (Figure S1B). Western‐blot analysis demonstrated downregulation of ERAP1 expression in HeLa cells with miR‐UL112‐5p (Figure 1A,C). Since a target for miR‐UL112‐5p in the ERAP1 3′UTR in mice is lacking, an ERAP1 shRNA was designed to achieve this objective (Figure 1B,D).

Experiments were performed to evaluate whether ERAP1 silencing affected transgene expression in commonly used target cells. The results indicated that when HeLa cells and human dendritic cells (hDCs) were transfected with pAAV‐OVA‐miRNA‐UL112‐5p, OVA expression exhibited no significant decrease compared with pAAV‐OVA (Figure 1E,F). In C2C12 and JASWII transfected with pAAV‐OVA‐ERAP1 shRNA, OVA expression did not decrease compared with that in pAAV‐OVA (Figure 1G,H). Our findings indicate that our vector can efficiently silence ERAP1 in target cells, including HeLa cells, muscle and APC, while maintaining a negligible effect on transgene expression.

### 3.2 ERAP1 Downregulation Inhibits Antigen Presentation

**FIGURE 2:**
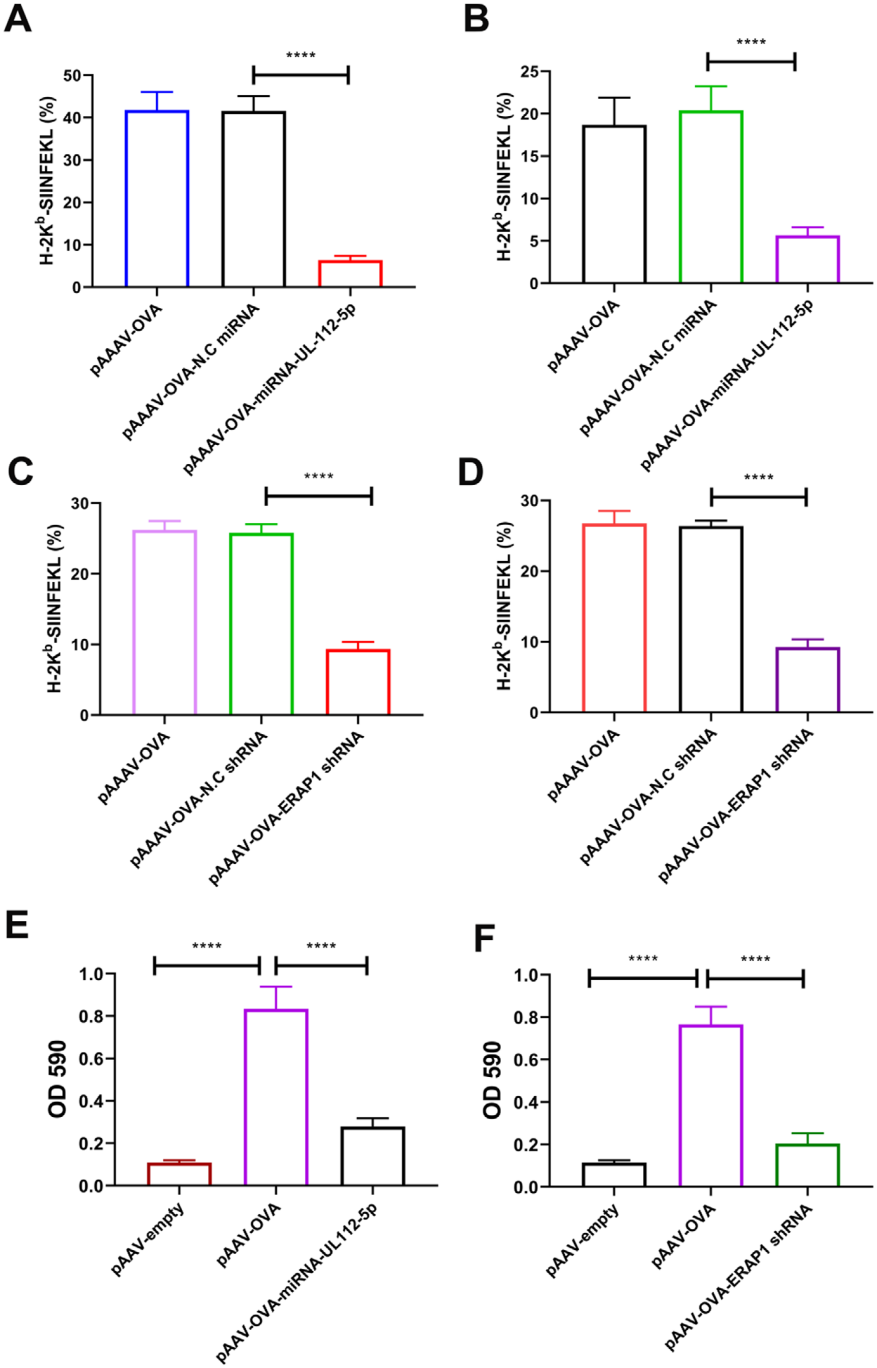
Effects of miRNA‐112, ERAP1 shRNA on OVA antigen presentation and DC‐induced T cells. The effect of pAAV‐OVA‐miRNA‐UL112‐5p transfection in HeLa‐K^b^ (A) and human DC (B) on antigenic peptide presentation at cell membrane was examined. The effect of pAAV‐OVA‐ERAP1 shRNA transfection of C2C12‐K^b^ (C) and JAWSII (D) on antigenic peptide presentation at cell membrane was examined. Human DCs (E) JAWSII (F) were transfected with pAAV‐empty, pAAV‐OVA‐miRNA‐UL112‐5p or pAAV‐OVA‐ERAP1 shRNA for 36 h, and co‐cultured using B3Z cells for 24 h, prior to LacZ activity measurement to explore B3Z activation. The results are indicated by mean ± SD (*n* = 3). ****p < 0.0001.

HeLa‐K^b^ and C2C12‐K^b^ cells stably expressing H‐2K^b^ were used to measure antigen presentation. 25‐D1.16 mAb specifically recognises the MHC‐I‐SIINFEKL complex, which is present on the cell membrane surface, while it does not recognise H‐2Kb bound to an unrelated peptide. Therefore, 25‐D1.16 monoclonal antibodies were used to detect the OVA antigen presentation. The results showed that when HeLa‐K^b^ and human DC were transfected with pAAV‐OVA or pAAV‐OVA‐scrambled miRNA plasmid, a large amount of SIINFEKL‐H‐2Kb was observed on the cell surface; however, the SIINFEKL antigenic peptide content on the membrane surface of cells transfected with pAAV‐OVA‐miRNA112‐5p was significantly reduced (Figure 2A,B). The findings from ERAP1 shRNA silencing on antigen presentation in mouse cells are consistent with those observed in human cells (Figure 2C,D).

In addition, an in vitro DC‐T cell co‐culture experiment was performed to measure the antigen presentation function of DCs. Human DCs or JAWSII were transfected for 36 h using pAAV‐OVA, pAAV‐OVA‐miRNAUL112‐5p, or pAAV‐OVA‐ERAP1 shRNA. After transfection, the cells co‐culture with B3Z for another 24 h. The results indicate that miRNA‐UL112‐5p and ERAP1 shRNA inhibited antigen presentation in DCs and thus inhibited activation in B3Z cells (Figure 2E,F).

### 3.3 rAAV‐OVA‐ERAP1 shRNA Transduction Stabilises OVA Expression and Reduces OVA Antibody Production In Vivo

**FIGURE 3:**
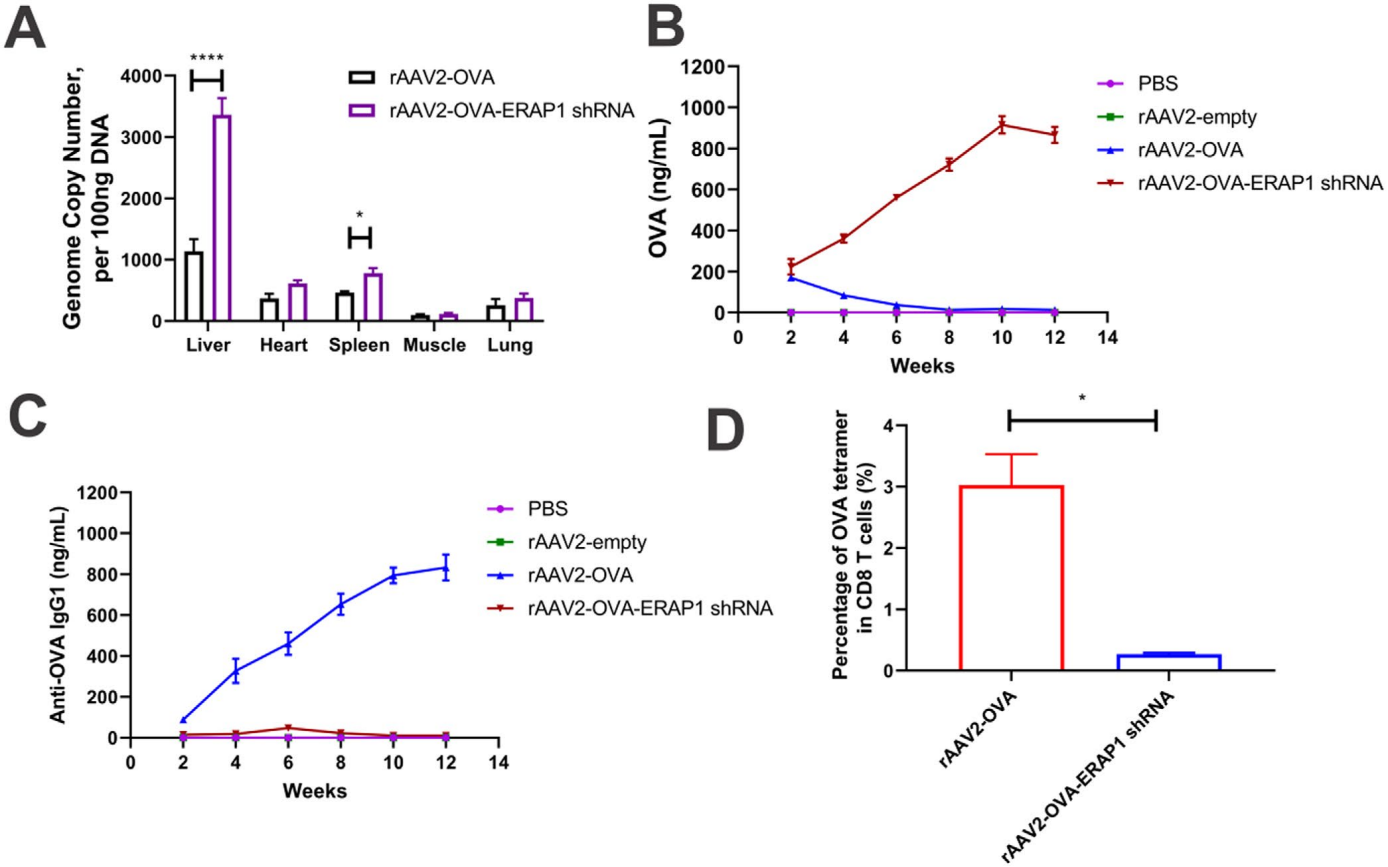
rAAV2‐OVA‐ERAP1 shRNA sustains OVA expression and inhibits the production of anti‐OVA IgG1 in vivo. rAAV2 vector delivery was performed on day 0 before the collection of serum at 2‐week intervals for 12 weeks. (A) Two weeks after intravenous (i.v.) delivery, qPCR assay on rAAV vector genome copies (GCs) in the liver, heart, spleen and muscle (*n* = 3). (B–C) ELISA analysis on circulating OVA (B) and anti‐OVA IgG1 levels (C) in animals given i.v. injection of rAAV2‐OVA or rAAV2‐OVA‐ERAP1 shRNA vectors (2 × 10^11^ GCs/mouse, *n* = 3). Serum was obtained over the course of 12 weeks. The results are indicated by mean ± SD (*n* = 3). (D) Anti‐CD8a/H‐2Kb SIINFEKL tetramer was added to stain OVA‐specific CD8+ T cells separated in the spleens from vector‐injected C57BL/6 mice, followed by flow cytometry. The data are indicated by mean ± SD (*n* = 3). *p* values were analysed upon unpaired *t‐*test. *p < 0.05, ****p < 0.0001.

The following experiment aimed to assess the influence of rAAV2‐OVA‐ERAP1 shRNA on transgene‐specific B cell responses in vivo. Therefore, intravenous injection was performed with PBS, rAAV2‐empty, rAAV2‐OVA, or rAAV2‐OVA‐ERAP1 shRNA into C57BL/6 mice. At two‐week post‐injection, more vector genomes were measured in the liver and spleen, while fewer vector genomes were measured in the lung and muscle (Figure 3A). The vector genome in the liver injected with rAAV2‐OVA‐ERAP1 shRNA was increased by 3.3 times. OVA protein and anti‐OVA antibody expression was monitored during 12 weeks (Figure 3B,C). Mice injected with rAAV2‐OVA‐ERAP1 shRNA exhibited high and persistent circulating OVA levels, and the anti‐OVA antibody response was negligible (IgG1). By contrast, mice injected with the rAAV2‐OVA showed significantly decreased OVA levels at 4 weeks of treatment and lost detectable OVA levels at 8 weeks after injection. This observation was accompanied by increasing levels of anti‐OVA IgG over time. In addition, rAAV2‐OVA‐ERAP1 shRNA transduced tissue exhibited increased vector genome compared with rAAV2‐OVA‐injected tissue at week 12 (Figure S2), suggesting an immune response resulting in the clearance of transduced tissue and the subsequent loss of vector genomes.

To further substantiate the OVA‐specific CD8+ T‐cell activity in normal and treated mice, MHC tetramer staining was performed. In this study, a tetramer of 4 H‐2Kb MHC class I molecules conjugated with phycoerythrin (PE) was employed to bind to the OVA peptide (SIINFEKL). After 2‐week rAAV delivery, OVA‐specific CD8+ T cells were significantly reduced in splenocytes separated from rAAV2‐OVA‐ERAP1 shRNA‐treated mice compared with the rAAV2‐OVA group (Figure 3D). This result suggests that the reduction in transgene epitope presentation in APCs is sufficient to suppress the CTL response. Together, these data suggest that mice treated by rAAV2‐OVA vector mounted immunity to the OVA that can be effectively circumvented by incorporating ERAP1 shRNA.

### 3.4 rAAV2‐OVA‐ERAP1 shRNA Reduced Costimulatory Signals With DCs and Inflammatory Cytokine Generation

**FIGURE 4:**
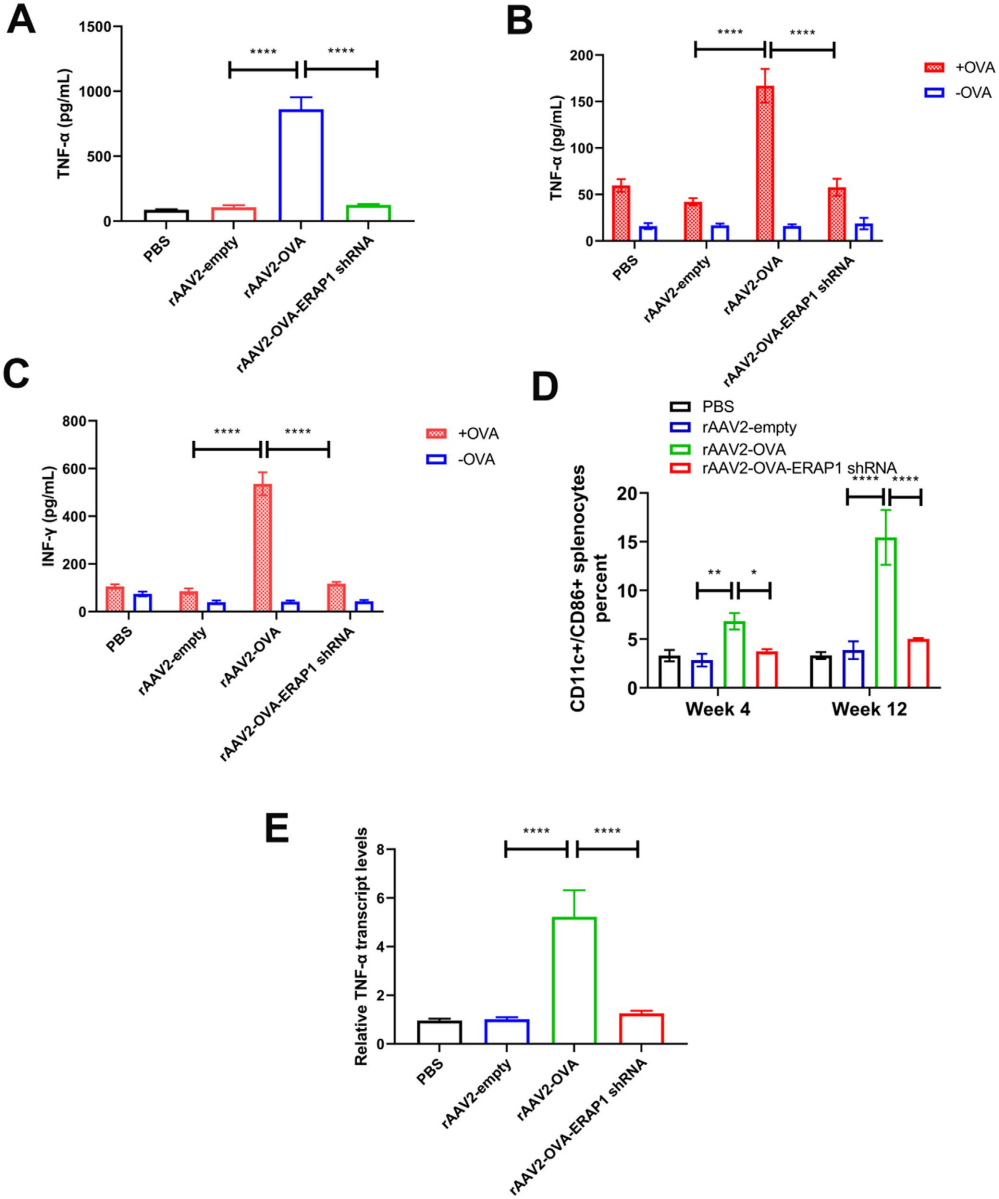
Effect of rAAV2‐OVA transgenic vector containing ERAP1 shRNA on reducing OVA‐specific T‐cell responses. (A) TNF‐α levels in serum were analysed using ELISA (mean ± SD, *n* = 3). *p* values were measured using ANOVA and Tukey's post hoc test. (B–C) TNF‐α and IFN‐γ responses upon 5 μg/mL OVA stimulation (+OVA) in splenocytes obtained in mice at 12‐week following rAAV injection. *p* values were measured by adopting ANOVA and Tukey's post hoc test. (D) The 6‐week‐old C57BL/6 mice were given i.v. injection of PBS, rAAV2‐OVA, rAAV2‐OVA‐ERAP1 shRNA, or rAAV2 empty vector (2 × 10^11^ GCs/mouse). CD11c+/CD86+ splenocytes collected in 4‐ and 12‐weeks following vector treatment were quantified through flow cytometry. Bar graphs indicate mean ± SD (*n* = 3). *p* values were obtained through ANOVA and *Holm‐Šídák's* post hoc test. (E) TNF‐α mRNA expression in entire spleens was quantified through RT‐qPCR. The data are indicated by mean ± SD (*n* = 3). *p* values were measured through ANOVA and Dunnett's post hoc test. *p < 0.05, **p < 0.01, ****p < 0.0001.

To assess the efficiency of antigen presentation following rAAV2‐OVA transduction and its impact on activating APCs, costimulatory factors produced by DCs were investigated. TNF‐α is a principal proinflammatory cytokine, exerting a vital role in both the innate and adaptive immune mechanisms [33, 34]. TNF‐α is generated through DCs and other immune cells, which can increase CD80/86 costimulatory molecule levels on DCs [34]. CD86 (B7‐2) interacts with CD28, which plays a vital role in the activation and survival of T cells [35]. After mice being treated with rAAV2‐OVA, the circulating TNF‐α level was increased by 8.6 times compared with rAAV2‐OVA‐ERAP1 shRNA group (Figure 4 A). Four weeks and 12 weeks after rAAV injection, splenocytes from injected PBS, rAAV2‐OVA, rAAV2‐OVA‐ERAP1 shRNA, or rAAV2‐empty mice were harvested. When exposed to OVA treatment, splenocytes collected from rAAV2‐OVA group at week 12 released large amounts of IFN‐γ and TNF‐α, suggesting a robust anti‐antigen anamnestic response. By contrast, the response was reduced in splenocytes isolated from the rAAV2‐OVA‐ERAP1 shRNA‐treated mice (Figure 4B,C). Similar IFN‐γ and TNF‐α levels were released in rAAV2‐OVA‐ERAP1 shRNA splenocytes compared to PBS and rAAV2‐empty vector splenocytes, conforming to the condition of cells collected at week 4 (Figure S3A,B).

This study aimed to examine the effects of rAAV2‐OVA‐ERAP1 shRNA treatment on CD86‐positive splenocytes, which are known to be a subset of DCs (a type of immune cell) exerting a vital impact on T‐cell activation. Splenocytes were collected at 4‐ and 12‐week post‐rAAV treatment, isolated using myeloid cell markers, including DCs (CD11c), and examined by flow cytometry to explore the CD86 level. The data indicated that the proportion of CD86‐positive splenocytes in rAAV2‐OVA‐ERAP1 shRNA‐treated mice significantly decreased relative to that in rAAV2‐OVA‐treated mice. The empty capsid group of rAAV2 exhibited a similar proportion of CD11c/CD86+ splenocytes as the PBS control group, and the differences were not significant (Figure 4D and Figure S4A). Moreover, this finding was consistent with the significantly decreased TNF‐α levels observed in the spleens of rAAV2‐OVA‐ERAP1 shRNA‐treated mice (Figure 4E and Figure S4B).

### 3.5 rAAV‐OVA‐ERAP1 shRNA Continuously Decreased Transgene Immunity After Redosing and Increased OVA Level

**FIGURE 5:**
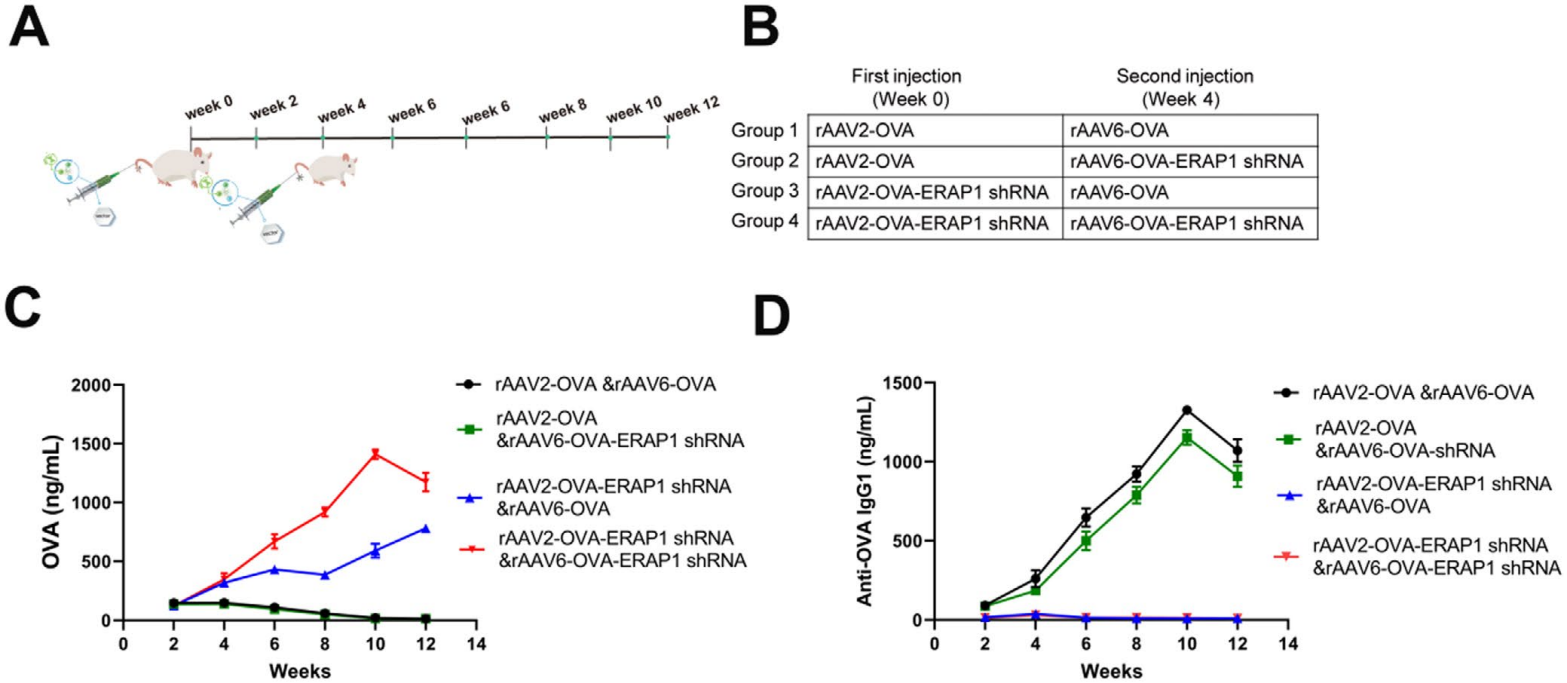
rAAV‐OVA‐ERAP1 shRNA allows for redosing and increases transgene levels. Timeline representing rAAV‐redosing assay (A) and depiction of rAAV serotype as well as OVA transgene in every injection (B). rAAV2‐OVA or rAAV2‐OVA‐ERAP1 shRNA (2 × 10^11^ GCs/mouse) were i.v. injected in C57BL/6 mice. rAAV6‐OVA or rAAV6‐OVA‐ERAP1 shRNA (2 × 10^11^ GCs/mouse) were i.v. injected in the above mice at 4 weeks following initial injection (*n* = 3). (C, D) ELISA analysis on circulating OVA (C) and anti‐OVA IgG levels (D) in vector‐injected mice. Serum was obtained within 12 weeks. Results are indicated by mean ± SD (*n* = 3).

A single dose of the rAAV‐OVA‐ERAP1 shRNA vector suppressed adaptive immunity against the transgene. Then, we aimed to evaluate whether the same effect could be achieved following repeated vector administrations. C57BL/6 mice were injected with rAAV2‐OVA or rAAV2‐OVA‐ERAP1 shRNA. Subsequently, a second round of vector injections was performed using AAV6 capsids instead of the previous AAV2 capsids (rAAV6‐OVA or rAAV6‐OVA‐ ERAP1 shRNA) after the initial injection 4 weeks (Figure 5A,B). The reason for using AAV6 capsids is serologically different from AAV2 capsids, which can therefore lower the risk of capsid‐specific immune responses interfering with our experimental outcomes [36]. Serum levels of OVA and anti‐OVA antibodies were also determined over 12 weeks. Our results suggested that animals administered an initial injection of rAAV2‐OVA‐ERAP1 shRNA and later injection of rAAV6‐OVA with/without ERAP1 shRNA generation increased and maintained OVA levels, while circulating anti‐OVA IgG1 levels could be ignored (Figure 5C,D). OVA expression was further increased when repeated administration of both rAAV2 and rAAV6 incorporated ERAP1 shRNA (Figure 5C, red tracks). By contrast, mice first dosed with rAAV2‐OVA showed near baseline serum OVA levels and high anti‐OVA IgG1 levels, regardless of whether the second injected rAAV co‐expression ERAP1 shRNA (Figure 5C,D).

### 3.6 rAAV‐OVA‐ERAP1 shRNA Inhibits CTL Responses and Reduces Transduced Cell Clearance

**FIGURE 6:**
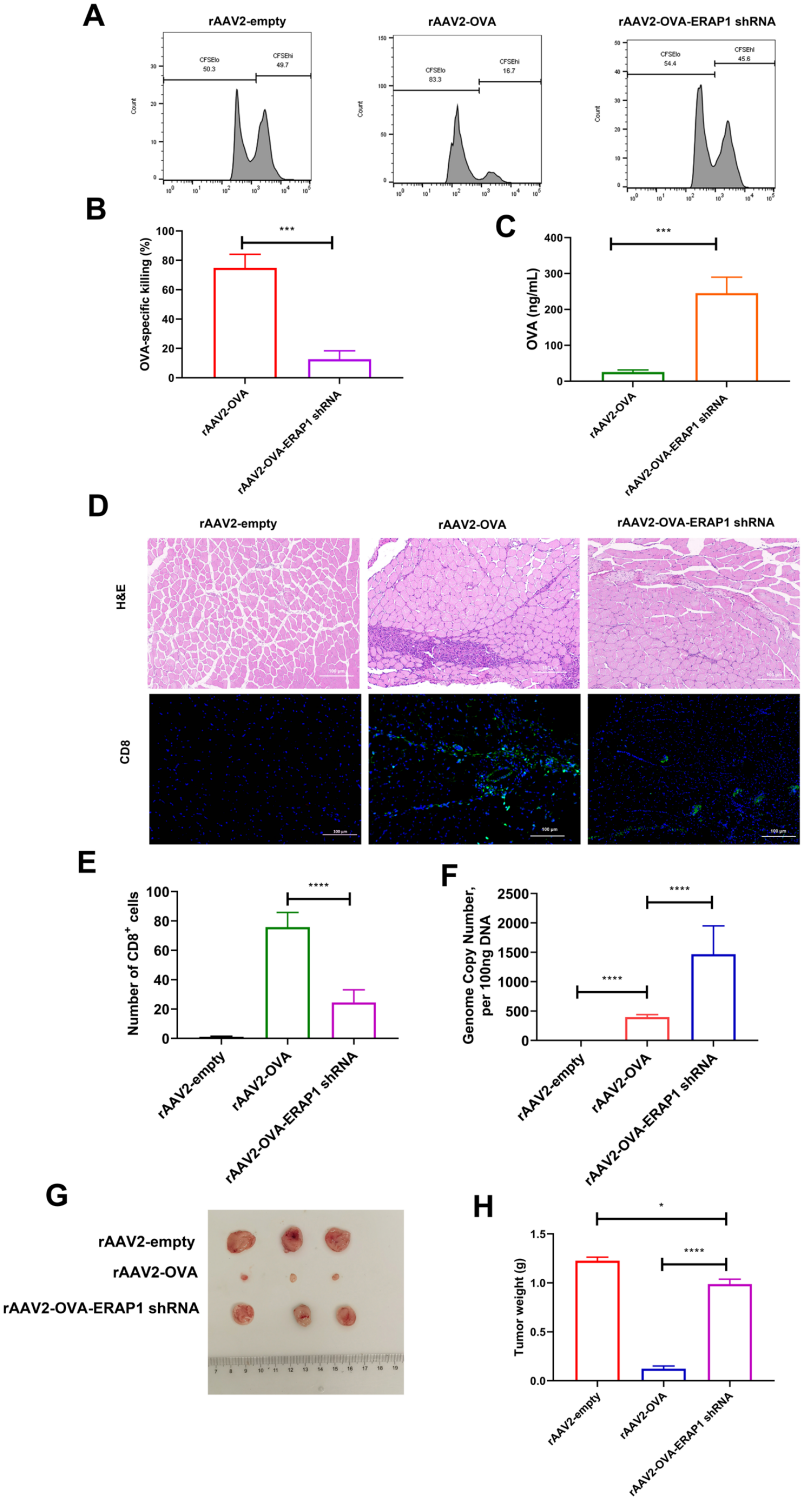
rAAV2‐OVA‐ERAP1 shRNA decreases CD8+ cell infiltration and OVA‐specific tissue elimination. Mice were treated with rAAV and a CTL assay was performed to investigate the effect of the treatment in vivo. (A) The 6‐week‐old C57BL/6 mice were i.v. injected with rAAV2‐empty, rAAV2‐OVA, or rAAV2‐OVA‐ERAP1 shRNA (2 × 10^11^ GCs/mouse). At 17 days post‐injection, high‐dose CFSE (CFSEhi, 5 μM) was added to label target cells in spleens in an identical strain of treatment‐naive mice, followed by pulsing using SIINFEKL peptide (OT‐1, 1 μg/mL); alternatively, low‐dose CFSE (CFSElo, 0.5 μM) was used to label target cells without pulsing. (B) Afterwards, target cells were blended in a 1:1 ratio and subsequently cotransferred intravenously (at a dosage of 2 × 10^7^ cells) to mice that had been treated either with rAAV2‐empty, rAAV2‐OVA or rAAV2 OVA‐ERAP1 shRNA. After 6 h, splenocytes were separated for flow cytometry, aiming to explore remaining target cell proportion. Data are indicated by mean ± SD (*n* = 3). ***p < 0.001, unpaired *t‐*test. (C) Serum OVA levels of rAAV2‐OVA or rAAV2‐OVA‐ERAP1 shRNA‐treated animals were examined through ELISA (mean ± SD, *n* = 3). ***p < 0.001, unpaired *t‐*test. (D) rAAV2‐OVA, rAAV2‐OVA‐ERAP1 shRNA, or AAV2‐empty vector (2 × 10^11^ GCs/mouse, *n* = 3) was given through i.m. injection into C57BL/6 mice. Muscle samples were obtained at 2 weeks post‐injection to perform H&E and CD8 staining. (E) CD8+ T‐cells proportion was quantified from 4 fields (×20 magnification). *p < 0.05, unpaired *t‐*test (*n* = 3). (F) qPCR analysis on rAAV vector genome copies in injected TA muscles; harvested 2 weeks post‐injection. Bar graphs indicate mean ± SD (*n* = 3). *P* values measured using ANOVA and Sidak's post hoc test. ***p < 0.001. (G) C57BL/6 mice receiving rAAV2‐OVA‐ERAP1 shRNA treatment exhibited decreased tumour killing following injection of E.G7‐OVA lymphoma cells. rAAV2‐OVA or rAAV2‐OVA‐ERAP1 shRNA (2 × 10^11^ GCs/mouse) were given through i.v. injection into C57BL/6 mice. At 2 weeks post‐injection, E.G7 cells with OVA expression were given into mice through subcutaneous inoculation (2 × 10^6^ cells/mouse, *n* = 3). Tumour tissue was collected at 15 days post‐injection. (H) Statistical analysis of tumour weight. *p < 0.05, ***p < 0.001, ****p < 0.0001; One‐way ANOVA combined with Tukey's test.

To evaluate the functional potency of OVA‐specific CD8^+^ T cells in spleens of mice injected with rAAV2‐OVA containing or lacking ERAP1 shRNA sequences, an in vivo CTL experiment was performed. This analysis examined functional CTLs in CFSE‐labelled and adoptively transferred target cells (Figure 6A). After 17 days of rAAV2 treatment, mice treated with rAAV2‐OVA cleared 74.8% of the target cells, and the OVA content was 25.6 ng/mL. However, mice injected with rAAV2‐OVA‐ERAP1 shRNA exhibited significantly reduced OVA‐specific target cell elimination (approximately 12.6%), the OVA content was significantly increased (245.5 ng/mL) (Figure 6B,C).

To more comprehensively understand the role of rAAV2‐OVA‐ERAP1 shRNA on transgene‐specific T‐cell response, this study analysed histopathology of rAAV‐injected muscle tissue and performed IHC analysis of CD8+ T cells at 2 weeks post‐treatment. H&E staining of tibialis anterior muscles injected with rAAV2‐OVA exhibited severe cellular infiltration. Immunohistochemical analysis indicated that CD8+ T cells infiltrates were predominant (Figure 6D,E). While muscle injected with rAAV2‐OVA‐ERAP1 shRNA showed only minor immune cell infiltration, a litter of CD8+ cells were present (Figure 6D,E). This finding suggests that con‐expression ERAP1 shRNA in OVA decreases transduced muscle fibre elimination due to a reduced CD8+ T‐cell infiltration. The results partially supported Figure 6F data and Figure 3A and also explained why vector genome copies were significantly decreased in rAAV2‐OVA‐treated livers compared with rAAV2‐OVA‐ERAP1 shRNA‐treated livers.

Subsequently, we examined the co‐expression of ERAP1 shRNA with OVA on OVA‐specific CTL function. C57BL/6 mice were subcutaneously injected with mouse T‐cell lymphoma cells (E.G7‐OVA) expressing OVA protein. The results demonstrated that mice treated with rAAV2‐OVA‐ERAP1 shRNA group had significantly larger tumours than those treated with rAAV2‐OVA group. This indicated that rAAV2‐OVA effectively suppressed tumour development by inducing an OVA‐specific CTL response. By contrast, rAAV2‐OVA‐ERAP1 shRNA inhibited T‐cell response specific to OVA, causing insufficient suppression of tumour development (Figure 6G,H).

## 4 Discussions

**FIGURE 7:**
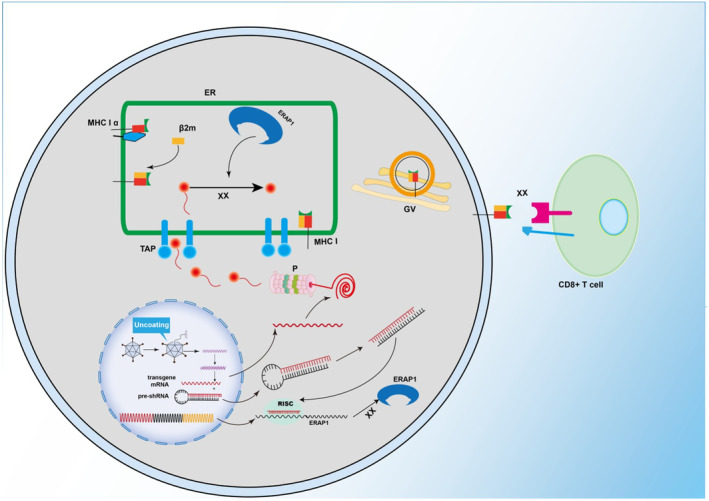
Schematic diagram of rAAV‐OVA‐ERAP1 shRNA inhibiting transgenic antigen presentation in cell. rAAV vector transfects APC through receptor‐induced endocytosis. Nuclear uncoating and endosomal escape can produce endogenous transgene product. The antigen after endogenous production in cytosol can be processed via proteosome, which can then enter ER through TAP, ERAP1, an endoplasmic reticulum resident protein, specifically cleaves antigen peptides to an 8‐10AA size for optimal binding to the MHC‐I. Additionally, it is expressed on MHC‐I in the ER and shuttled between Golgi apparatus and cell surface through ‘direct presentation’. When the transgene contains ERAP1 shRNA or miRNA‐UL112‐5p, these mechanisms can suppress ERAP1 expression. Therefore, the antigenic peptide delivered into ER via TAP cannot be further cleaved, thus preventing its presentation to the APC cell surface. P, proteosome; TAP, transporter associated with antigen processing; ER, endoplasmic reticulum; ERAP1, endoplasmic reticulum aminopeptidase 1; GV, golgi vesicle; MHC, Major histocompatibility complex; RISC, RNA‐induced silencing complex.

In this study, we designed a novel rAAV vector that can silence ERAP1 by co‐expressing miRNA‐UL‐112‐5p or ERAP1 shRNA to effectively evade the immune response to transgene products. Figure 7 presents its specific mechanism. During viral infection, ERAP1 exerts a vital role in the establishment of immunity against specific epitopes. ERAP1 depletion in mice significantly impacts the peptide pool during the process of mouse cytomegalovirus (CMV) and lymphocytic choriomeningitis virus (LCMV) infection and CD8+ T‐cell responses against LCMV‐derived antigens [37, 38, 39]. In ERAP1‐KO mice, many unstable, probably N‐terminal‐extended, MHC class I‐bound peptides are present on the cell surface [40]. Previous findings in ERAP1‐KO mice indicate that ERAP1 downregulation influences the generation of numerous HCMV‐derived antigen peptides during viral infection, thus preventing recognition of viral antigens via CD8+ T cells during the process of host immunity [41]. Our experimental results demonstrated that the use of miRNA‐UL112‐5p or ERAP1 shRNA to decrease the expression of ERAP1 significantly decreased the presentation of the OVA antigen peptide SIINFEKL on the surface of HeLa‐K^b^. OVA antigen presentation on APC is inhibited, therefore affecting T‐cell activation (Figure 2).

Recently, findings have revealed that both humoral and cellular immunity to intramuscular injection of rAAV‐encoded transgene products has been detected [8, 42, 43, 44]. The generated CD8 T cells can cause a CTL response that can disrupt and damage transduced cells, influencing transgene expression and therapeutic efficacy. The produced antibodies can reduce transgene expression, further affecting therapeutic efficacy. Currently, researchers employ two primary strategies incorporating specific promoters or miRNA binding sequences in the transgene expression cassette to prevent immune responses induced by transgene products. Desmin promoter or synthetic C5‐12 promoter may provide transgene expression predominantly in muscle, which can significantly reduce the immune response; however, there is a degree of leakage in DCs and this leakage may still induce an immune response to transgene [8, 45, 46]. Integrating binding sites for one or more miRNAs (miRNABS) into the 3’‐UTR of highly immunogenic OVA cDNA can prevent transgene immunity when delivered by rAAV while maintaining expression in muscle [45, 47, 48]. Unfortunately, muscle still showed a significant immune response, probably because miRNABS did not appear to be sufficient to block the expression of OVA in APC cells or to completely hydrolyse the binding sequence of OVA mRNA. The above two methods are strategies which can be used for reducing transgene expression in APC cells. In addition, we used ERAP1 shRNA or miRNA‐UL‐112‐3p to silence ERAP1, a protein that is critical in antigen presentation. This method is suitable for inhibiting APC cells from presenting antigens through the MHC class I molecular pathway.

Previous research indicates that ERAP1 is an essential factor in the immune system's ability to recognise virus‐infected cells, as demonstrated by multiple results, including escape mutation emergence that impairs ERAP1 activity, causing a diminished CD8+ T‐cell response [26, 49]. Previous study also confirms that AAV2‐LacZ i.m. injection cannot provide adequate DC transduction or antigen presentation to facilitate the adaptive response [50]. As expected, higher doses of AAV vectors generally not only produce higher levels of cellular immunity to the antigens they encode, but may also induce capsid‐induced immune responses [7, 51]. Whether miRNA‐UL112‐5p or ERAP1 shRNA co‐expression with transgene can inhibit the immune response induced by rAAV capsid needs to be further investigated.

Muscle and liver, the terminally differentiated tissues, have already been identified as the primary targets used in long‐term, in vivo rAAV transduction. However, due to host cell turnover, the non‐replicating and episome rAAV genomes are probably lost over time. Therefore, the development of effective therapeutic strategies for diseases may require repeated administration of vectors. Based on our redosing studies, rAAV2‐OVA‐ERAP1 shRNA injection effectively inhibited cell‐regulated immunity, even when treatment re‐administration was needed (Figure 5C,D). Furthermore, the effectiveness of ERAP1 shRNA or miRNA‐UL112‐5p mediated suppression of transgenic immunity was dependent on cells or tissues transduced by rAAV. Preclinical studies have suggested that recombinant AAV vectors can sustainably express a multitude of both non‐self and self‐transgenes in tissues, including muscle, liver, lung, brain, heart and eye [7, 17, 52, 53, 54]. The developed rAAV‐OVA‐ERAP1 shRNA or rAAV‐OVA‐miRNA‐UL112‐5p platform may also suppress immunity induced by non‐self‐transgene products.

Cross‐presentation has been widely suggested to be vital for CD8+ T‐cell responses against secreted proteins [55]. However, our research findings demonstrate that suppressing antigen expression in APCs is enough to diminish CD8+ T‐cell responses and prevent antibody responses against the transgene products. Importantly, co‐expression ERAP1shRNA was sufficient for attenuating the clearance of transduced muscle and E.G7‐OVA xenographs. Co‐expression of miR‐UL112‐5p or ERAP1 shRNA to prevent transgene‐specific immunity may be beneficial for certain rAAV gene therapy applications.

The potential immunotoxicity of AAV vectors is primarily attributed to cytotoxic T lymphocytes initiated by AAV capsid proteins [18, 56] or transgene [17, 57]. However, there is no definitive approach for circumventing these immunotoxicity in future clinical applications. The data presented in this study indicate that the co‐expression of either miRNA‐UL112‐5p or ERAP1 shRNA into the transgene can effectively inhibit the immune response induced by the rAAV transgen products, regardless of whether gene transfer is achieved through intramuscular injection or intravenous injection. The emergence of high‐dose rAAV‐related fatal incidents in clinical practice has prompted researchers to focus on capsid‐induced immune response [51]. In the future studies, our team will concentrate on investigating the effect of rAAV‐OVA‐miRNA‐ERAP1 shRNA on capsid‐induced immune responses. If the developed rAAV‐ERAP1 shRNA can successfully suppress capsid‐induced immunity, it will significantly facilitate the development of rAAV based therapeutic strategies.

## Author Contributions

**Xiaoping Huang:** conceptualization (equal), data curation (equal), writing – original draft (equal), writing – review and editing (equal). **Xiao Wang:** conceptualization (equal), data curation (equal), writing – original draft (equal). **Yaqi Sun:** data curation (equal). **Xinrui Xie:** data curation (equal). **Luming Xiao:** data curation (equal). **Yihang Xu:** data curation (equal), formal analysis (equal). **Qiongshi Yan:** data curation (equal), formal analysis (equal). **Xianxiang Xu:** data curation (equal), software (equal). **Ling Li:** data curation (equal). **Wentao Xu:** data curation (equal). **Wenting Weng:** data curation (equal), methodology (equal). **Wenlin Wu:** data curation (equal). **Xiaolan Xie:** formal analysis (equal), resources (equal), supervision (equal). **Congjie Dai:** data curation (equal), resources (equal), software (equal), supervision (equal). **Yong Diao:** conceptualization (equal), writing – original draft (equal), writing – review and editing (equal).

## Ethics Statement

Experiments involving animal subjects followed the ARRIVE guidelines and the Guide for Care and Use of Animals in Research of the People's Republic of China. The Huaqiao University (Quanzhou, China) Scientific Ethics Committee authorised all the experimental protocols (no. AHQU019326).

## Consent

The authors have nothing to report.

## Conflicts of Interest

The authors declare no conflicts of interest.

## Supporting information


Figure S1.
Figure S2.
Figure S3.
Figure S4.

## Data Availability

The data supporting the findings of this study are available within the article.
