# Supplementary material for: Effective Reduction of Transgene‐Specific Immune Response With rAAV Vectors Co‐Expressing miRNA‐UL112‐5p or ERAP1 shRNA
Source: J Cell Mol Med. 2025 Jan 17;29(2):e70308. doi: 10.1111/jcmm.70308 (PMC11740984; doi:10.1111/jcmm.70308)
Supplement: Supplementary file 1 — Figure S1. Figure S2. Figure S3. Figure S4. [file JCMM-29-e70308-s001.docx]

**Supporting Information**


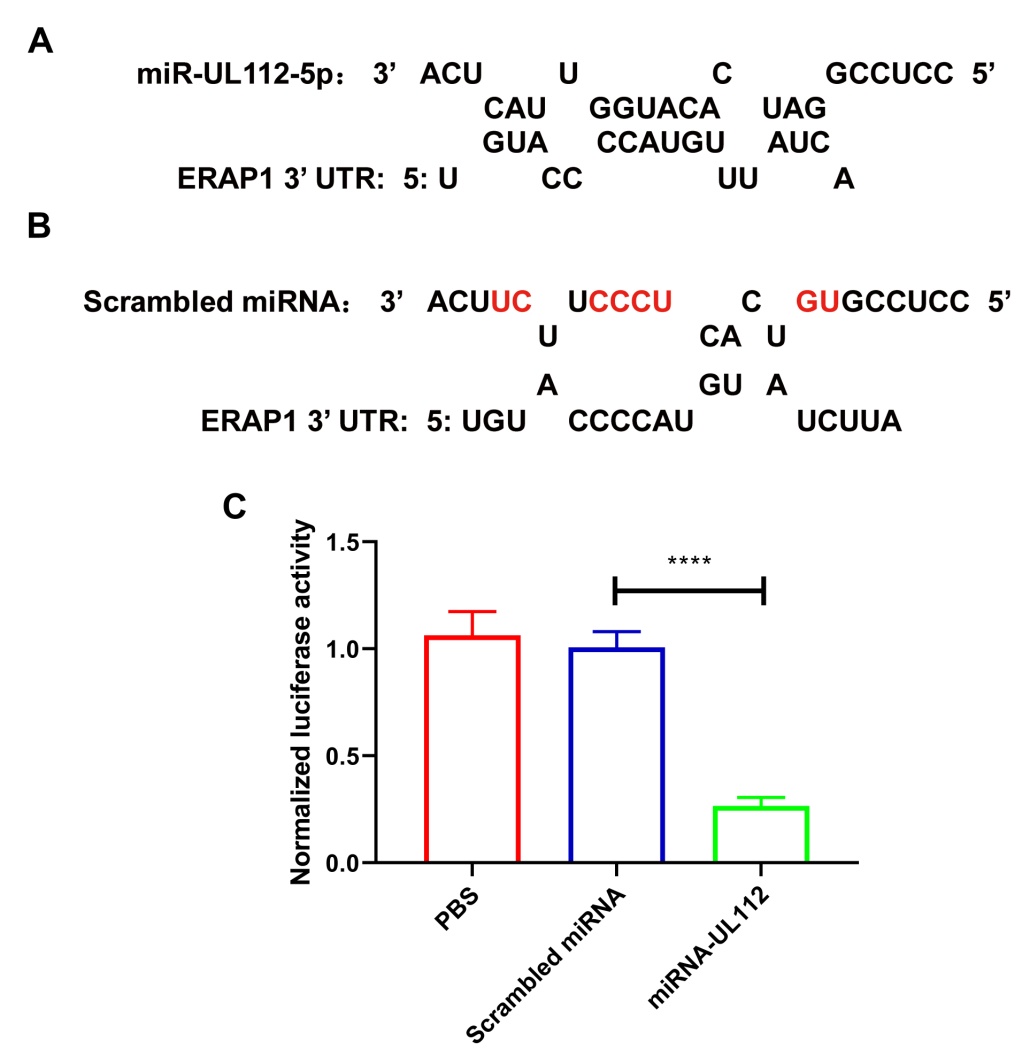


FIGURE S1 MiRNA-UL112-5p targets ERAP1 mRNA by binding to the ERAP1 3’-UTR. (A) miRNA-UL112-5P binding sites in ERAP1 (GenBank ID: AF222340.1) 3’UTR predicted by Targetscan 5.2, Luciferase activity was inhibited in the presence of miRNA-UL112-5p (A) compared with that of Scrambled miRNA (B). Relative luciferase activity in the HeLa cells 48h after transfection of psiCHECK-ERAP1-3'UTR-Luc (B) and miRNA-UL-112-5p or scrambled miRNA (C). Data are shown as mean ± SD of three independent assays, ****P < 0.0001.


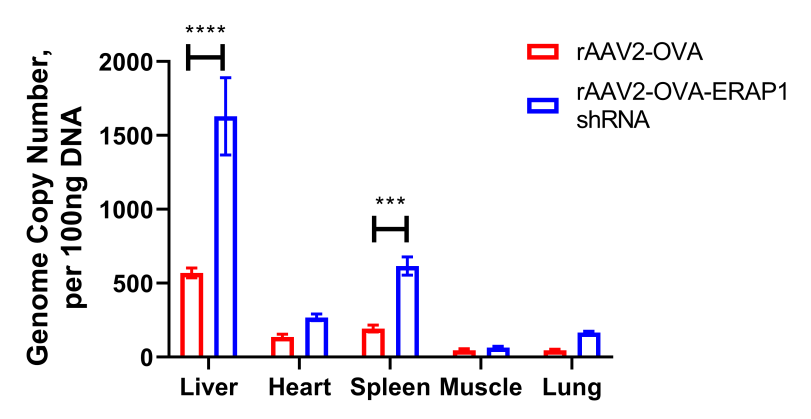


FIGURE S2. Inclusion of ERAP1 shRNA elements into the OVA transgene results in high levels of vector genomes detected in tissues of mice. 12 weeks after intravenous (i.v.) delivery, qPCR assay on rAAV vector genome copies (GCs) in the liver, heart, spleen, and muscle (n=3). Bar graphs represent mean ± SD (n = 3). P values determined by ANOVA with Sidak’s post-hoc test. ***p < 0.001, ****p < 0.0001.


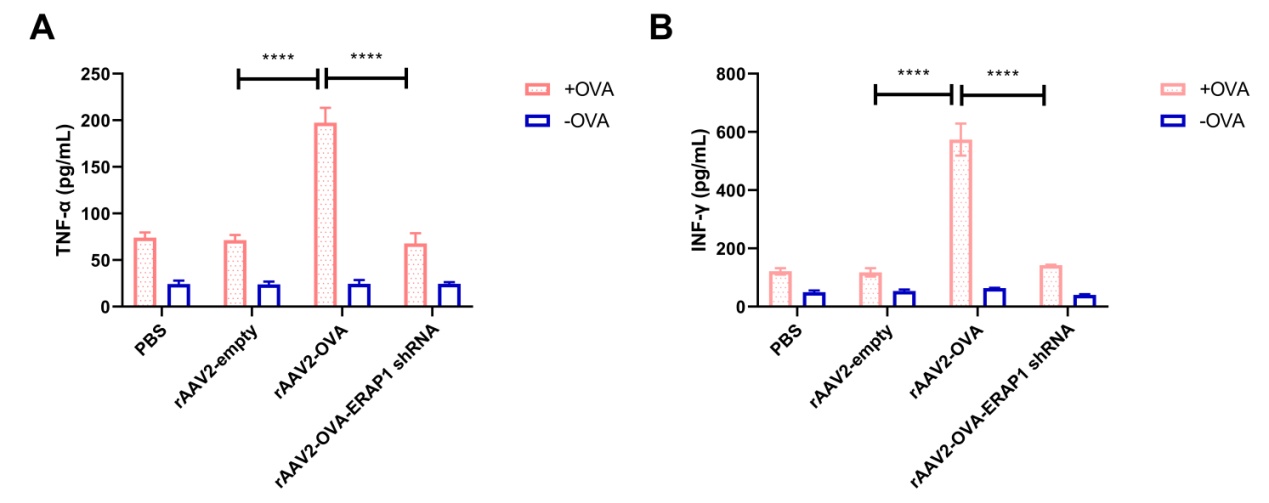


FIGURE S3. OVA-induced upregulation and ERAP1 shRNA-mediated attenuation of TNF-d and INF-γ expression is due to dendritic cell populations. (A-B) Assessment of IFN-γ and TNF-α response to OVA protein (+OVA, 5 μg/mL) by splenocytes isolated from mice four weeks post-vector injection. Three days after treatment, supernatants were collected and quantitated by ELISA (mean ± SD, n = 5). P values determined by ANOVA with Tukey’s post-hoc test) -OVA, mock treatment.


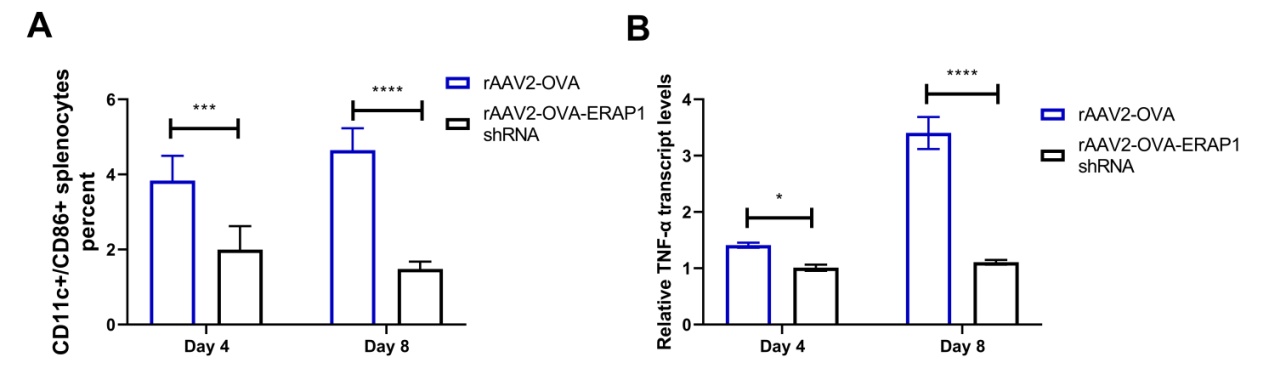


FIGURE S4. ERAP1 shRNA incorporation suppresses transgene immunity by reduction of activated T cells and inflammatory cytokines. (A) Six-week-old C57BL/6 male mice were injected intramuscularly with PBS, rAAV2-OVA, rAAV1-OVA-ERAP1 shRNA, or rAAV2 empty vector (2 × 10^11^ GC/mouse, n = 3). Quantification of CD11c+/CD86+ splenocytes harvested 4 or 8 weeks after vector administration by flow cytometry. (B) Quantitation of TNF-α mRNA levels in whole spleens by RT-qPCR. *p < 0.05, ***p < 0.001, ****p < 0.0001, unpaired t test (n = 3).
